# Emodin Prevents Intrahepatic Fat Accumulation, Inflammation and Redox Status Imbalance During Diet-Induced Hepatosteatosis in Rats

**DOI:** 10.3390/ijms13022276

**Published:** 2012-02-20

**Authors:** Anna Alisi, Anna Pastore, Sara Ceccarelli, Nadia Panera, Daniela Gnani, Giovannella Bruscalupi, Mara Massimi, Giulia Tozzi, Fiorella Piemonte, Valerio Nobili

**Affiliations:** 1 Liver Unit of “Bambino Gesù” Children’s Hospital, IRCCS, Rome 00165, Italy; E-Mails: sara.ceccarelli@opbg.net (S.C.); nadia.panera@opbg.net (N.P.); daniela.gnani@yahoo.it (D.G.); nobili66@yahoo.it (V.N.); 2 Laboratory of Biochemistry, of “Bambino Gesù” Children’s Hospital, IRCCS, Rome 00165, Italy; E-Mail: anna.pastore@opbg.net; 3 Department of Biology and Biotechnology “C. Darwin”, “La Sapienza” University, Rome 00185, Italy; E-Mail: giovannella.bruscalupi@uniroma1.it; 4 Department of Basic and Applied Biology, University of L’Aquila, L’Aquila 67010, Italy; E-Mail: mara.massimi@univaq.it; 5 Neuromuscular and Neurodegenerative Disease Unit, “Bambino Gesù” Children’s Hospital, IRCCS, Rome 00165, Italy; E-Mails: giulia.tozzi@opbg.net (G.T.); fiorella.piemonte@opbg.net (F.P.)

**Keywords:** hepatosteatosis, emodin, high fat diet, high fructose diet, redox status

## Abstract

High-fat and/or high-carbohydrate diets may predispose to several metabolic disturbances including liver fatty infiltration (hepatosteatosis) or be associated with necro-inflammation and fibrosis (steatohepatitis). Several studies have emphasized the hepatoprotective effect of some natural agents. In this study, we investigated the potential therapeutic effects of the treatment with emodin, an anthraquinone derivative with anti-oxidant and anti-cancer abilities, in rats developing diet-induced hepatosteatosis and steatohepatitis. Sprague-Dawley rats were fed a standard diet (SD) for 15 weeks, or a high-fat/high-fructose diet (HFD/HF). After 5 weeks, emodin was added to the drinking water of some of the SD and HFD/HF rats. The experiment ended after an additional 10 weeks. Emodin-treated HFD/HF rats were protected from hepatosteatosis and metabolic derangements usually observed in HFD/HF animals. Furthermore, emodin exerted anti-inflammatory activity by inhibiting the HFD/HF-induced increase of tumor necrosis factor (TNF)-α. Emodin also affected the hepatocytes glutathione homeostasis and levels of the HFD/HF-induced increase of glutathionylated/phosphorylated phosphatase and tensin homolog (PTEN). In conclusion, we demonstrated that a natural agent such as emodin can prevent hepatosteatosis, preserving liver from pro-inflammatory and pro-oxidant damage caused by HFD/HF diet. These findings are promising, proposing emodin as a possible hindrance to progression of hepatosteatosis into steatohepatitis.

## 1. Introduction

Hepatosteatosis or simple fatty liver is characterized by accumulation of fat in liver cells. There are several different causes of hepatosteatosis, including chronic alcohol consumption, B and C viral hepatitis, type 2 diabetes, obesity and some metabolic aberrations [1,2]. Actually, nonalcoholic fatty liver disease (NAFLD) is considered the most prevalent form of hepatosteatosis associated with obesity and metabolic syndrome [3,4]. During the last 20 years, NAFLD has reached worrying proportion involving 20–30% of adults and 3–10% of children in Western countries [5]. NAFLD genesis is multifactorial and comprises different patterns of liver injuries including simple hepatosteatosis alone or in combination with nonalcoholic steatohepatitis (NASH), with or without fibrosis [6].

In recent years, many studies have provided new insights explaining potential mechanisms responsible for the switch from hepatosteatosis to NASH. So far it has been established that NAFLD pathogenesis and progression depends on different “hits” and it has been shown that the genetic makeup and dietary intake play key roles as leading factors [7,8]. A working model [9] has been proposed consisting of two sequential “hits”, the first conducting to the hepatic steatosis and the second towards the hepatic necro-inflammation determining the NASH condition and possibly fibrosis. Firstly, the insulin resistance (IR), and/or the derangement of fatty acid metabolism (*de novo* lipogenesis, lower beta oxidation, impairment of triglyceride clearance and the diminished export of very-low-density lipoprotein), leads to hepatic fat accumulation and increased liver sensitivity to other possible subsequent hits [3,10]. Followed by a still largely unknown mechanism, multifactorial complex interactions have been described as responsible for the “second hit” leading to the more advanced form of NASH which can possibly predispose to cirrhosis [11,12]. This further hit includes oxidative stress, lipid peroxidation, imbalance of inflammatory cytokines and adipokines and augmentation of pathogen- or damage-associated molecular patterns [13–15].

Nowadays the intervention against the NAFLD status encompasses two different and complementary directions: lifestyle changes and/or pharmacological treatment against specific hits potentially involved in NAFLD pathogenesis (*i.e.*, insulin resistance and oxidative stress) [16]. In the last decade many noteworthy efforts have been made for ameliorating the hepatic damage in NAFLD. It has been extensively demonstrated that metformin, vitamin E or placebo treatments do not have positive effects on liver injury although vitamin E is able to improve the hepatocellular ballooning degeneration [17].

The current known targets for treatment of NAFLD are limited in number and are not even sufficiently defined and a breakthrough for new tolerated and efficient compounds is needed [16]. In fact, many studies are aimed at testing the effect of natural agents on NAFLD evolution [18–25]. It has been proven that Silibinin (silybin), a polyphenolic molecule constituent of silymarin (a flavonolignan extracted from *Silibum marianum),* has anti-oxidant and hepatoprotective effects [20]. Moreover, it protects against cirrhosis, decreases fibrosis if complemented to vitamin E and phospholipids and decreases both insulin resistance and plasma markers of liver fibrosis in NAFLD patients [21]. Curcumin, a polyphenol and an active component of turmeric (*Curcuma longa*), is another natural compound investigated by several laboratories. Clinical studies showed a protective action against fructose-induced hepatic steatosis by improving inflammation, hyperlipidemia, reducing insulin resistance and interrupting leptin signaling [22–24]. Interestingly, it has been demonstrated that emodin (1,3,8-trihydroxy-6-methylanthraquinone), which is an active herbal component traditionally used in China for treating a variety of diseases, might have a role in the disease regression in NAFLD-induced rats. In fact, emodin significantly decreased the body weight, liver index, serum activities of ALT, blood lipids, hepatic triglyceride and considerably improved the hepatic histology features [25]. Despite these encouraging results, to date no further thoughtful studies have been made to understand mechanisms and reliability of emodin in NAFLD models.

The wide diffusion of NAFLD in developed countries and its close correlation with cirrhosis, place the study of both prevention and therapeutic approaches, based on natural safe and efficient agents, in a central position of interest. Thus, in this study we attempted to investigate the potential preventive properties of emodin in a diet-induced hepatosteatosis in rats.

## 2. Results and Discussion

### 2.1. Effects of Five Weeks HFD/HF Diet on Rats

Nowadays the pivotal role of fructose in NAFLD pathogenesis is widely recognized [26,27]. In fact, the excessive fructose intake may enhance the synthesis of triacylglycerols that accumulate in the liver causing hepatosteatosis, and trigger the inflammatory response that lead to NASH [28,29]. Noteworthy, recently Kohli *et al*. [30] developed a model of NAFLD that well resembled human disease by using an animal model fed with high-fructose medium-chain-trans-fat diet. Moreover, more recently we developed another interesting model of NAFLD that combined high fat diet with high 30% fructose-enriched drinking water [31]. In this study, we used the same high fat/high fructose (HFD/HF) dietetic regimen compared to standard diet (SD) for 5 and 15 weeks. At the end of the 5th week of treatment, SD and HFD/HF animal body weight data was recorded. At the same time, blood samples were collected from caudal vein to perform metabolic analysis. As shown in Table 1, animal body weight displayed an increase of about 19% compared to the beginning of diet protocols. However, no sign of NAFLD was already evident in HFD/HF, as shown by the absence of statistically relevant changes in body weight and metabolic parameters between the two groups of treatment. These results suggest that rats may accumulate significant traits of hepatosteatosis if the treatment with HFD/HF diet is extended for a longer period of time. In fact, as we recently demonstrated, a 3 months treatment with HFD/HF diet is necessary to develop hepatosteatosis and NASH in Sprague-Dawley rats [31].

### 2.2. Effects of Emodin on Body Weight, Liver Weight and Metabolic Parameters in HFD/HF Rats

Dong *et al*. [25], as reported above, demonstrated the emodin therapeutic action in Sprague-Dawley rats fed with high-caloric diet for 12 weeks. Whereas, here, we evaluated the preventive effect of emodin (40 mg/kg/day) in 5 weeks pre-treated rats that received the treatment with SD or HFD/HF for an additional 10 weeks. Data collected from these animals were compared with two groups subjected to SD or HFD/HF without addition of emodin. At the end of treatments, the body and liver weight, and liver index (liver weight/body weight × 100), were evaluated. As reported in Table 2, in HDF/HF group the weight patterns were significantly increased compared with those in the SD group (*P* < 0.05). Interestingly, in HFD/HF animals emodin treatment caused a slight increase of body weight (*P* < 0.05), that was counteracted by a significant decrease of liver weight and index (*P* < 0.01). As expected, HFD/HF diet resulted in a significant rise in plasma levels of ALT, triglycerides, insulin and glucose, and HOMA-IR (*P* < 0.01). Interestingly, emodin treatment in HFD/HF group considerably reduced metabolic parameters bringing their values at levels very similar to those observed in SD animals (see Table 2).

### 2.3. Hepatoprotective and Anti-Inflammatory Effects of Emodin in HFD/HF Rats

**Figure 1:**
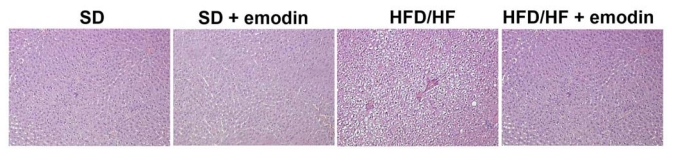
Histological changes of rat liver in each group stained by H-E (Magnification 200×).

Currently, the diagnosis of NAFLD, and particularly the identification of steatohepatitis, is based on the histological evaluation of the liver biopsy [6]. Therefore, liver histology, despite its limitations, is the most reliable method to assess grading and staging of all histological features that characterize NASH both in humans and animal models [32,33]. Here, we analyzed the histological pattern of NAFLD by Hematoxylin-Eosin (H-E) staining. Liver of HFD/HF animals showed typical microvacuolar and macrovacuolar steatosis, ballooning, and some inflammatory cells, confirming the successful establishment of the animal model. With the emodin treatment HFD/HF animals displayed reduced cytological steatosis and ballooning, and a complete absence of inflammatory cells (Figure 1). These results are in agreement with previous studies demonstrating the hepatoprotective role of emodin both in the case of hepatosteatosis and in other liver diseases [34–36]. Emodin exerts multiple effects, including anti-proliferative, anti-cancer, anti-inflammatory and hepatoprotective activities [37–39]. Emodin has also been reported to reduce serum hyaluronic acid, laminin expression, hepatic levels of hydroxyproline and the degree of liver fibrosis [40].

**Figure 2:**
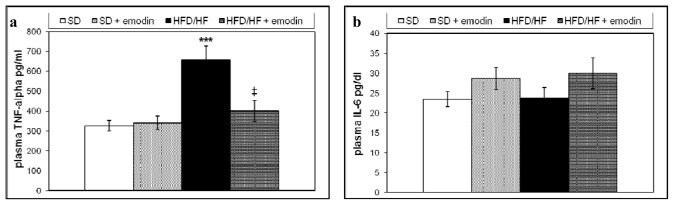
Plasma levels of TNF-α (**a**) and IL6 (**b**) in all groups of treatment. Values are means ± SD. *** *P <* 0.001, *vs*. SD group. ‡ *P <* 0.01, *vs*. HFD/HF group.

In this, study we also evaluated the systemic anti-inflammatory properties of emodin by the analysis of plasma levels of two relevant pro-inflammatory cytokines in NAFLD, tumor necrosis factor (TNF)-α and interleukin 6 (IL6) [41]. Although, the increased hepatic expression of TNF-α and IL6 has been described in NAFLD obese patients, changes and significance of the circulating levels of these cytokines still remain unclear [42,43]. Recently, it has been demonstrated that either high carbohydrate diet or high fat diet are able to increase plasma levels of TNF-α in mice [44]. In the present study, we examined the circulating levels of TNF-α and IL6 in all animal groups. As reported in Figure 2a and b, HFD/HF regimen induced a significant increase in the plasma levels of TNF-α with respect to the SD; whereas no significant differences in the IL6 plasma levels were found between the two groups. Interestingly, the treatment with emodin impeded the rise of plasma TNF-α, maintaining this circulating cytokine at levels similar to those observed in SD group.

Anti-inflammatory activity of emodin has already been reported, but this study represents the first evidence demonstrating its potential preventive action on systemic inflammation occurring in NAFLD.

### 2.4. Emodin Promotes Recovery of Redox Status Imbalance in Primary Hepatocytes from HFD/HF Rats

**Figure 3:**
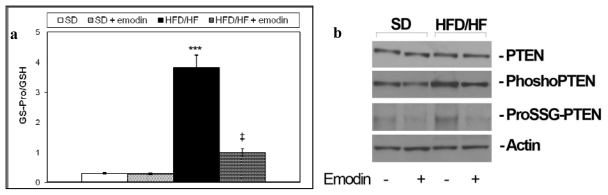
(**a**) ProSSG/Tot GSH ratios were reported. Histograms are the mean value ± S.D. *** *P <* 0.001, *vs*. SD group. ‡ *P <* 0.01, *vs*. HFD/HF group; (**b**) Western blotting of total, phosphorylated and glutathionylated PTEN in primary hepatocytes isolated from livers of each group of treatment.

Glutathione is a tripeptide that exists in a reduced (GSH) and oxidized form (GSSG). The ratio between these two glutathione forms is fundamental for maintaining the redox status balance and important cellular functions, such as cell proliferation [45]. GSSG may also occur as protein-bound glutathione (ProSSG). ProSSG plays a pivotal role in the regulation of important regulatory proteins including NFκB and PTEN (glutathionylation) [46,47]. We recently demonstrated that HFD/HF diet was able to promote redox status imbalance particularly increasing the ratio between ProSSG and total GSH (Tot GSH = GSSG + GSH + ProSSG) in primary hepatocytes [48]. Here we confirmed this data and demonstrated that emodin treatment protects from the increment of ProSSG/Tot GSH ratio in primary hepatocytes isolated from HFD/HF (Figure 3a). These results corroborate the hypothesis of a strong anti-oxidant action of emodin on steatotic livers. Moreover, as in the previous published study [48], we found that HFD/HF diet caused an increment in phosphorylation/glutathionylation of hepatic PTEN, that is consistent with the inhibition of its activity; here we assayed if emodin was able to protect primary hepatocytes from PTEN phosphorylation/glutathionylation. As reported in Figure 3b, emodin treatment preserves PTEN either from phosphorylation and glutathionylation.

A quantity of data has shown that the alterations of PTEN expression and activity are related with liver disorders and its deregulation plays a key role both in hepatic insulin sensitivity and the onset of steatosis, steatohepatitis and fibrosis [49]. Moreover PTEN regulates the PI3K/Akt signaling pathways [50] and Akt is differently involved in glucose homeostasis and diabetes [51]. In this study we have demonstrated that the increased PTEN phosphorylation/glutathionylation in HFD/HF diet rats may be counteracted by emodin treatment. The significance of these results is intriguing since the recovery of PTEN activation should explain the improved effect on insulin resistance in emodin-treated HFD/HF rats. Therefore, in the future, it would be interesting to study the activity of PTEN before and after the emodin treatment. Moreover, it would be worth investigating a possible Akt role in the mechanism regulating the positive effect of emodin with regard to the disrupted glucose homeostasis in NAFLD rat model.

### 2.5. Emodin Protects HFD/HF Primary Hepatocytes Rats from Further Oxidative Stress Damage

**Figure 4:**
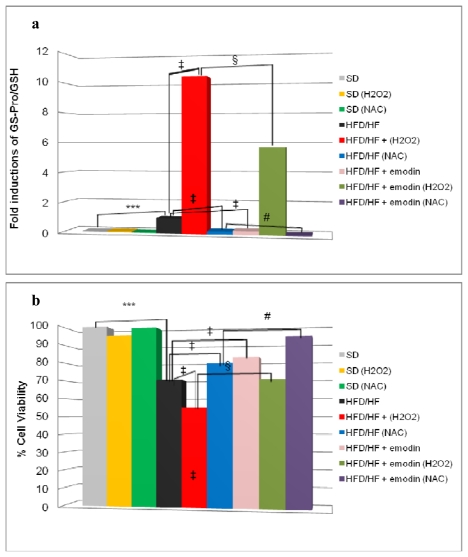
(**a**) ProSSG/Tot GSH ratios were reported as fold induction; (**b**) Cell viability at 24 h was evaluated by a neutral red assay and reported as percentage compared with the control (SD). Histograms are the mean value ± S.D. *** *P <* 0.001; ‡ and § *P <* 0.01, # *P <* 0.05.

Hepatosteatosis may evolve to NASH with fibrosis that destructs liver tissue integrity and cell homeostasis. This phenomenon occurs by a pool of secondary hits, among which the most relevant is the oxidative stress [13]. Our hypothesis is that preventive treatment with emodin not only may protect HFD/HF rats from hepatosteatosis, but it may also induce an anti-oxidant stable reaction of hepatocytes against further oxidative stress damage and predispose liver cells to a better response to additional anti-oxidant conventional drugs (e.g., *N*-acetylcysteine and α-tocopherol). To assess this hypothesis, we isolated primary hepatocytes from rats treated with SD, or HFD/HF regimen with or without emodin. These cells were cultured for 24 h in the presence or the absence of the following treatments: 500 μM hydrogen peroxide (H_2_O_2_) or 1 mM *N*-acetylcysteine (NAC). At the end of the experiment, we collected cells to evaluate ProSSG/Tot GSH ratio and cell viability. As shown in Figure 4a, the treatment with H_2_O_2_ dramatically increased ProSSG/Tot GSH ratio in hepatocytes from HFD/HF rats, but this effect was significantly reduced in hepatocytes from emodin-treated HFD/HF animals. On the other hand, in hepatocytes from HFD/HF rats, the treatment with NAC caused a relevant decrease of ProSSG/Tot GSH ratio that was enhanced by the concomitant presence of emodin in animals’ *in vivo* treatment.

Finally, cell viability results indicated that hepatocytes from HFD/HF animals displayed a reduced cell viability, after 24 h culture, compared with SD-derived hepatocytes (Figure 4b). However, in hepatocytes from HFD/HF, this reduced viability was significantly counteracted by NAC treatment and retrieved even more if the hepatocytes were derived from emodin treated HFD/HF rats as described above.

Altogether, these findings indicate that HFD/HF regimen may profoundly alter oxidative stress response and cell homeostasis of hepatocytes, but these effects are counteracted by the preventive treatment with emodin.

## 3. Experimental Section

### 3.1. Animals and Primary Hepatocytes

Twenty-four male Sprague–Dawley rats (120–140 g) were obtained from Harlan Italy (San Pietro al Natisone, UD, Italy). The animals received treatment in agreement with the European guidelines of the local committee for animal care and welfare. The animals used in this study were part of a large experimental protocol approved by Italian Ministry of Health. They were located in plastic cages under standard conditions with free access to water and food, at the Certified Animal Facility of the University of Rome, “La Sapienza”. The animals were fed with standard rat chow for 5 days then equally grouped based on two different dietetic regimens: a standard diet (SD) and a high-fat/high-fructose diet (HFD/HF). SD contained 5% of energy derived from fat, 18% from proteins, and 77% from carbohydrates (3.3 kcal/g), while HFD/HF contained 58% of energy derived from fat, 18% from protein, and 24% from carbohydrates (5.6 kcal/g; Laboratorio Dottori Piccioni, Gessate Milano, Italy); plusfructose (30%) that was added to the drinking water. After 5 weeks half of SD and HFD/HF were treated with emodin (40 mg/kg/day) from Sigma-Aldrich, Milan, Italy. Fluid and food intake were assessed every two-days at the replacement. We found no significant differences between consumption of food and water among the groups.

Randomly after 6 h fasting, from each group of animal, liver tissues were taken for biochemistry and histology. Further, from each group primary hepatocytes have been isolated using a perfusive method as previously described [52]. Briefly, the rats were anesthetized by intraperitoneal administration of sodium pentobarbital (5 mg/100 g body weight). The liver was perfused firstly with a calcium-free Hank’s balanced salt solution containing 2% BSA and 0.6 mM ethyleneglycotetraacetic acid, and secondly with Hank’s solution containing 4 mM calcium chloride and 0.04% collagenase. Liver cells were released into a Krebs–Henseleit buffer with 2% BSA. The hepatocytes were seeded on collagen-coated plates at density between 1.5 × 10^4^ and 3 × 10^4^/cm^2^. After 24 h from plating hepatocytes from SD, HFD/FD, SD + Emodin, HFD/HF + Emodin animals were subjected to following treatments: 10 μL PBS (NT) or *N*-acetylcysteine 1mM (NAC) or H_2_O_2_ 500μM (H_2_O_2_). Hepatocytes were harvested 24 h later, centrifuged and collected for the experiments.

### 3.2. Biochemical Determinations and Inflammatory Markers

Blood samples obtained from caudal vein after 6 h fasting were collected in sterile glass tubes containing 0.15% EDTA. Blood samples were centrifuged at 3000 for 15 min to obtain plasma. Plasma samples were immediately used to perform enzymatic and photocolorimetric assay to determine the levels of alanine aminotransferase (ALT), triglycerides, total cholesterol, glucose and insulin. Enzymatic and colorimetric assays were performed using standard procedures as indicated by kits purchased from different companies: ALT assay kit from Randox Laboratories Ltd (Antrim, UK), triglycerides and cholesterol assay kits from Cayman Chemical (Ann Arbor, MI, USA), glucose assay kit from Abcam Inc (Cambridge, MA, USA), and rat insulin enzyme immunoassay kit from SPI-BIO (France). At 14 weeks, insulin resistance was calculated according to the homeostasis model assessment of insulin resistance (HOMA-IR) calculation: fasting plasma insulin (μU/mL) × fasting plasma glucose (mmol/L)/22.5. ELISA-based kits were used to assay the circulating levels TNF-α (Peprotech, Rocky Hill, New Jersey, USA) and IL6 (R&D Systems, Abingdon, UK).

### 3.3. Immunohistochemistry

Liver was fixed in 4% buffered formalin and embedded in paraffin. A measure of 3–5 μm sections were stained with haematoxylin and eosin (Bio-Optica, Milan, Italy). Then the specimens were evaluated under 10 × 20 light microscopic fields.

### 3.4. High-Performance Liquid Chromatography of GSH

The tissues and primary hepatocytes from liver mouse model NASH diet-induced or NASH diet-induced treated with Emodin were sonicated (Sonics Vibra Cell, Sonics & Material Inc., Newtown, CT, USA), three times for 2 s in 0.1 mL of 0.1 M potassium phosphate buffer (pH 7.2). Following the sonication levels of total (GSH Tot), reduced (GSH), oxidized (GSSG) and protein-bound (ProSSG) glutathione were analyzed by HPLC. HPLC equipment and conditions for analyzing the several forms of glutathione have been reported [53].

### 3.5. Immunoprecipitation and Western Blotting

Liver tissues were lysed in ice-cold Ripa buffer containing 50 mM Tris pH 7.5, 150 mM NaCl, 1% Triton X-100, 1 mM EGTA, 1% sodium deoxycholate and phosphatases 10% cocktail protease inhibitors. For the immunoprecipitation protocol of the glutathionylated proteins see previous published work [46]. Then protein extracts were resolved on 10–12.5% SDS-PAGE, transferred and immobilized onto nitrocellulose membrane (Amersham, Germany), blocked with 5% nonfat dry milk and incubated with appropriate primary and secondary antibodies. The anti-PTEN, anti-pPTEN, primary antibodies were purchased from Santa Cruz Biotech (CA, USA). Immunoblots were detected with the ECL system (Amersham) and the relative intensities of the specific bands were determined by densitometric analysis and referring to beta-actin protein expression.

### 3.6. Cell Viability

Cell viability was determined by a simple vital stain method that evaluates the accumulation of the neutral red dye in the lysosomes of viable, uninjured cells [54]. The simple vital Neutral red (Sigma-Aldrich) was dissolved in culture medium and added to cells for 1 h. The pH of the neutral red solution was adjusted in all the experiments to 6.35 with the addition of 1 M KH_2_PO_4_. Then, cells were washed thrice with PBS, and 1 mL of elution medium (EtOH/AcCOOH, 50%/1%) was added followed by gentle shaking for about 10 minutes to obtain the complete dissolution. Measures were acquired with spectrophotometer at 540-nm of absorbance.

### 3.7. Statistical Analysis

The results are reported as means ± SD. for at least four independent experiments. Statistical differences were determined by Student’s *t* test considering *P* < 0.05 as statistically significant.

## 4. Conclusions

In summary, in this study we reported for the first time the preventive effect of emodin on hepatosteatosis-dependent metabolic derangement and liver cell injury. In particular, our results demonstrated that emodin was able to protect rats, treated with high fat/high fructose diet, from insulin resistance, hypertriglyceridaemia, histological damage, systemic necro-inflammation, and oxidative stress. Furthermore, interestingly, we demonstrated that emodin treatment conferred to HFD/HF hepatocytes an important defense from additional oxidative stress, and an improved ability to react to classical anti-oxidant agents. Our data suggested that PTEN could be a target of emodin, but the full comprehension of the existing molecular mechanisms of this natural agent requires further study.

In conclusion, all these findings suggest the use of emodin, not only as a potential preventive agent in NAFLD diet-induced and a promising agent for hampering the progression to NASH, but also as a natural coadjuvant of the more classical antioxidant therapy.

## Figures and Tables

**Table 1:** Body weight and biochemical parameters at 5th week.

Parameters	SD	HFD/HF
Body weight (g)	140.8 ± 25.8	143.6 ± 23.2
Triglycerides (mg/dL)	92 ± 13.5	104 ± 19.4
Total cholesterol (mg/dL)	39.5 ± 5.8	44.0 ± 6.2
ALT (U/L)	22.3 ± 3.6	25.4 ± 4.5
Glucose (mg/dL)	65.0 ± 7.5	73.4 ± 10.1
Insulin (ng/mL)	0.23 ± 0.04	0.25 ± 0.03
HOMA-IR	0.92 ± 0.08	1.13 ± 0.15

Values are means ± SD.

**Table 2:** Body weight and biochemical parameters after emodin treatments for 10 weeks.

Parameters	SD	HFD/HF	SD+emodin	HFD/HF+emodin
Body weight (g)	295.6 ± 34.2	355.6 ± 28.7 *	308.1 ± 30.5	393.6 ± 31.4 †
Liver weight (g)	11.2 ± 1.3	15.2 ± 1.5 *	12.0 ± 0.5	12.4 ± 0.9 †
Liver weight/Body weight	3.7 ± 0.11	4.27 ± 0.09 *	3.8 ± 0.19	3.2 ± 0.2 ‡
Triglycerides (mg/dL)	105.1 ± 18.6	138.0 ± 16.3 **	110.4 ± 13.1	109.4 ± 15.7 ‡
Total cholesterol (mg/dL)	45.7 ± 7.2	48.5 ± 8.1	39.9 ± 10.2	48.0 ± 12.3
ALT (U/L)	25.2 ± 5.0	37.4 ± 3.9 **	26.0 ± 4.4	25.9 ± 5.8 ‡
Glucose (mg/dL)	69.4 ± 4.4	81.9 ± 5.7 **	68.3 ± 7.2	70.8 ± 9.6 ‡
Insulin (ng/mL)	0.24 ± 0.05	0.41 ± 0.09 **	0.25 ± 0.07	0.26 ± 0.03 ‡
HOMA-IR	1.02 ± 0.05	2.07 ± 0.18 **	1.05 ± 0.06	1.13 ± 0.19 ‡

Values are means ± SD.

* *P =* 0.05,

** *P <* 0.01, *vs*. SD group; Values are means ± SD.

† *P =* 0.05,

‡ *P <* 0.01, *vs*. HFD/HF group.
